# A phase I clinical trial evaluating the effect of food on the pharmacokinetics of TSL-1502, a glucuronide prodrug of a novel oral poly (ADP-ribose) polymerase inhibitor, in healthy Chinese subjects

**DOI:** 10.3389/fphar.2026.1786220

**Published:** 2026-04-10

**Authors:** Jing Zhou, Xiaolan Mi, Hongtao Liu, Rui Liu, Hong Zhang, Yanhua Ding

**Affiliations:** 1 Phase I Clinical Research Center, The First Hospital of Jilin University, Jilin, China; 2 Jiangsu Tasly Diyi Pharmaceutical Co., Ltd., Tianjin, China; 3 Changchun Jirun Jingyue Hospital, Changchun, Jingyue, China

**Keywords:** food effect, pharmacokinetics, poly (ADP-ribose) polymerase (PARP) inhibitor, safety, TSL-1502

## Abstract

**Objective:**

Poly (ADP-ribose) polymerase (PARP) inhibitors, based on synthetic lethality, have demonstrated significant efficacy in tumors with homologous recombination repair (HRR) deficiencies but are limited by severe hematological toxicities. TSL-1502 is a glucuronide prodrug of a novel oral small-molecule PARP inhibitor that releases its active metabolite, TSL-1502M, specifically in tumor tissues. This phase I study aimed to evaluate the impact of food on the pharmacokinetics and safety of TSL-1502, guiding its clinical use.

**Methods:**

Twenty healthy Chinese subjects were randomized into two groups (A and B, n = 10 each). In a two-period crossover design, participants received a single 200 mg oral dose of TSL-1502 under fasting or fed conditions. Plasma concentrations of TSL-1502 and TSL-1502M were measured using liquid chromatography-tandem mass spectrometry, and safety was assessed throughout.

**Results:**

Food delayed the absorption of TSL-1502 but did not significantly affect its elimination half-life. The least-squares geometric mean ratios (fed/fasted) for AUC_0-t_ and ${\text{AUC}}_{0-\infty }$ of TSL-1502 were 86.53% (71.23%–105.11%) and 88.36% (73.41%–106.35%), respectively. For TSL-1502M, the ratios were 90.03% (72.88%–111.22%) and 105.51% (93.51%–119.06%). Postprandial administration reduced TSL-1502 AUC_0-t_ by 13.47%, ${\text{AUC}}_{0-\infty }$ by 11.64%, and Cmax by 56.58%. For TSL-1502M, AUC_0-t_ decreased by 9.97%, ${\text{AUC}}_{0-\infty }$ increased by 5.51%, and Cmax decreased by 47.77%. All adverse events were grade I or II.

**Conclusion:**

Food delayed the absorption of TSL-1502 and substantially reduced its peak plasma concentration (Cmax), while AUC was moderately affected. TSL-1502 was well tolerated, and food did not affect its safety profile. These results suggest that TSL-1502 can be administered with or without food.

## 1 Introduction

Defects in the DNA damage response (DDR) pathway lead to genomic instability, thereby promoting tumor initiation and progression. DDR deficiencies not only drive tumorigenesis but also provide potential therapeutic targets for cancer treatment (Li et al., 2023; Lord and Ashworth, 2012; Jeggo et al., 2016; Roos et al., 2016; Pilie et al., 2019; O’Connor, 2015). Inhibition of key proteins within DDR pathways induces the accumulation of DNA damage in cancer cells, ultimately leading to cell death (Pilie et al., 2019). Synthetic lethality represents the only clinically approved strategy for DDR-targeted therapy. Based on this concept, poly (ADP-ribose) polymerase (PARP) inhibitors suppress PARP enzymatic activity in tumor cells harboring homologous recombination repair (HRR) gene defects (such as BRCA1/2 mutations), resulting in persistent DNA damage and subsequent cell death (Li et al., 2023; Huang et al., 2020; Ashworth and Lord, 2018; Guo et al., 2011). PARP inhibitors have been approved for the treatment of multiple cancers, including breast, prostate, and pancreatic cancers, and represent the most clinically advanced class of DDR inhibitors (Pilie et al., 2019; Curtin and Szabo, 2020).

Olaparib, the first PARP inhibitor approved and the most extensively studied to date, was approved in 2014 for the treatment of relapsed ovarian cancer in patients with BRCA mutations (Deeks, 2015). To date, at least seven PARP inhibitors (olaparib, rucaparib, niraparib, talazoparib, pamiparib, fluzoparib, and senaparib) have been marketed worldwide. These agents are generally used as maintenance therapies, significantly prolonging progression-free survival (Li et al., 2023; Pilie et al., 2019; Liu et al., 2023; Lee, 2025). Despite their clinical benefits, approximately 40%–70% of patients eventually develop resistance, limiting therapeutic efficacy (Kim and Nam, 2022). Moreover, hematologic toxicities—including anemia, neutropenia, and thrombocytopenia—resulting from simultaneous inhibition of PARP-1 and PARP-2 reduce the feasibility of combining PARP inhibitors with chemotherapy or other therapies, thereby restricting their clinical applications (Liu et al., 2023; Leo et al., 2018). Consequently, the development of novel PARP inhibitors with improved efficacy and safety is of great clinical importance.

TSL-1502, developed by Jiangsu Tasly Diyi Pharmaceutical Co., Ltd., is a glucuronide prodrug of a novel and potent oral small-molecule PARP inhibitor and employs an innovative glucuronide prodrug strategy, whereas currently approved PARP inhibitors are all administered in their pharmacologically active small-molecule forms rather than as prodrugs (Tranoy-Opalinski et al., 2014; Bruin et al., 2022). TSL-1502 is a β-glucuronidase-responsive prodrug that preferentially accumulates in tumors due to the high expression of this enzyme in the tumor microenvironment. β-Glucuronidase is released by necrotic tumor cells and infiltrating inflammatory cells, hydrolyzing TSL-1502 to release the active drug TSL-1502M, while remaining largely inactive in normal tissues where enzyme activity is low (Tranoy-Opalinski et al., 2014; Wang et al., 2021). This low-toxicity prodrug combined with tumor-specific enzymatic activation strategy enables increased drug concentration in tumors, reduced systemic exposure, and selective killing of malignant cells with improved safety profile. Following oral administration, plasma exposure of TSL-1502M accounted for approximately 0.3% and 17% of the parent compound in rats and dogs, respectively, indicating that systemic exposure is predominantly driven by the parent drug. This exposure profile may help maintain pharmacological efficacy while minimizing the risk of systemic toxicity. TSL-1502M undergoes further metabolism via both oxidative and conjugative pathways. Oxidative metabolism is primarily mediated by CYP2D6, whereas glucuronidation is catalyzed by multiple UGT isoforms, including UGT1A1, UGT1A3, UGT1A7, UGT1A8, UGT1A9, UGT1A10, and UGT2B15. Preclinical studies demonstrated that the drug is eliminated predominantly as unchanged parent via urinary and biliary excretion, and no additional pharmacologically active circulating metabolites have been identified (data on file). In preclinical studies, TSL-1502M demonstrated approximately 10-fold greater inhibitory potency against PARP1 enzymatic activity than olaparib (IC50: 0.66 ± 0.05 nM vs. 6.54 ± 0.01 nM) and showed superior antitumor efficacy with a favorable toxicity profile (Wang et al., 2021). Currently, a phase I clinical study in China (CTR20191039) is evaluating the safety, tolerability, and pharmacokinetics of TSL-1502 in patients with advanced solid tumors, while a randomized, active-controlled phase II study (NCT05420779) is investigating its efficacy and safety in HER2-negative advanced breast cancer patients with germline BRCA mutations.

To date, no data have been reported regarding the effect of food on the pharmacokinetics of oral TSL-1502 capsules. As with all orally administered drugs, assessing the potential impact of food on pharmacokinetic parameters is critical for establishing rational dosing recommendations. This phase I clinical trial evaluated the effects of food on the pharmacokinetics and safety of a single 200 mg oral dose of TSL-1502 capsules in healthy Chinese subjects, thereby providing evidence to guide subsequent clinical use.

## 2 Materials and methods

### 2.1 Study design

This study employed a single-center, randomized, open-label, two-period crossover design to assess the impact of food on the pharmacokinetics of a single 200 mg oral dose (50 mg × 4 capsules) of TSL-1502 capsules in healthy Chinese subjects, as well as the safety of TSL-1502 in this population. A total of 20 subjects were enrolled and randomly assigned to either Group A (fasting–fed) or Group B (fed–fasting), with 10 subjects in each group, maintaining a male-to-female ratio of 1:1. Subjects underwent screening within 14 days prior to drug administration. Eligible subjects underwent baseline assessments on Day-1 and were admitted to the clinical research center the day before dosing. In Group A, subjects received the first dose under fasting conditions after at least 10 h of overnight fasting on Day 1, with 200 mg of TSL-1502 (50 mg × 4 capsules) administered with approximately 240 mL of warm water. After a 7-day washout period, subjects entered the second period and were administered TSL-1502 after a high-fat meal. A high-fat meal was consumed 30 min prior to drug administration. The meal provided 800–1,000 kcal, with approximately 50% of calories derived from fat, and 150, 250, and 500–600 kcal derived from protein, carbohydrates, and fat, respectively. In Group B, subjects received the first dose after a high-fat meal, and after a 7-day washout period, entered the second period, during which they were administered TSL-1502 under fasting conditions. The remaining procedures for Group B were identical to those for Group A. Subjects were instructed to refrain from drinking water within 1 h before dosing and within 2 h post-dose. Lunch was provided 4 h post-dose, and dinner was provided 10 h post-dose. Each subject was hospitalized for 5 days during each study period, and remained seated for 4 h following drug administration.

The study protocol and informed consent documents were approved by the Ethics Committee of The First Hospital of Jilin University, and the study was conducted in accordance with the principles of the Declaration of Helsinki, the Good Clinical Practice (GCP) guidelines, and all applicable regulatory and legal requirements in China. Informed consent was obtained from all subjects prior to screening. The study was prospectively registered in the Chinese Clinical Trial Registry (chinadrugtrials.org.cn) under the registration number CTR20211575 on 2 July 2021 (https://www.chinadrugtrials.org.cn/).

### 2.2 Subjects

Healthy male and female Chinese subjects were enrolled if they met the following inclusion criteria: age between 18 and 50 years; body mass index (BMI) ranging from 18 to 28 kg/m^2^; and willingness to use effective contraception from screening through 6 months following the last dose of the study drug.

The main exclusion criteria included: smoking more than five cigarettes per day within the 3 months prior to screening; history of substance abuse or alcohol dependence; use of prescription drugs, over-the-counter medications, Chinese medicine, or dietary supplements within 2 weeks prior to screening or during the study; special dietary requirements that could interfere with the study diet; blood donation or loss of ≥100 mL within 1 month prior to screening; clinically significant diseases (including but not limited to gastrointestinal, renal, hepatic, neurological, hematological, endocrine, oncological, pulmonary, immunological, psychiatric, or cardiovascular diseases) within 12 months prior to screening that, in the investigator’s judgment, may affect the pharmacokinetics or safety assessment of the study drug; and clinically significant abnormal findings in electrocardiograms (ECGs) or laboratory tests.

### 2.3 PK analysis

Pharmacokinetic samples were collected at the following time points before and after the administration of TSL-1502 capsules in each period: 0 h (within 30 min before dosing), and 0.25, 0.5, 1, 2, 3, 4, 5, 6, 7, 8, 10, 12, 13, 14, 24, 36, 48, and 72 h post-dose. A total of 19 blood samples were collected per period, with approximately 3 mL of blood drawn into blood collection tubes containing K_2_-EDTA as an anticoagulant at each time point. Blood samples were processed within 60 min of collection by centrifugation at 2 °C–8 °C for 10 min at 1,500 ± 10 g. The upper plasma layer was then separated under frozen conditions and stored at −80 °C until analysis. All biological samples were analyzed at the Shanghai Institute of Materia Medica, Chinese Academy of Sciences using a validated, sensitive, and specific liquid chromatography-tandem mass spectrometry (LC-MS/MS) method for the quantification of TSL-1502 and its active metabolite, TSL-1502M, in plasma. The method validation report is kept at the Shanghai Institute of Materia Medica, Chinese Academy of Sciences. For TSL-1502, the calibration curve was linear over the range of 1.00–500 ng/mL with a lower limit of quantification (LLOQ) of 1.00 ng/mL. Intra-day precision was ≤3.4% (≤5.9% at LLOQ) and inter-day precision was ≤3.3% (≤6.2% at LLOQ). Intra-day accuracy ranged from −4.4% to 1.7% (−7.1%–1.3% at LLOQ), and inter-day accuracy ranged from −4.1% to 0.1% (−1.9% at LLOQ). For TSL-1502M, the calibration curve was linear over 0.100–50.0 ng/mL with an LLOQ of 0.100 ng/mL. Intra-day precision was ≤4.3% (≤9.2% at LLOQ) and inter-day precision was ≤3.4% (≤9.8% at LLOQ). Intra-day accuracy ranged from −3.1% to 3.8% (−1.6%–10.3% at LLOQ), and inter-day accuracy ranged from −2.7% to 2.0% (3.5% at LLOQ). All validation parameters met regulatory acceptance criteria.

### 2.4 Safety assessments

Safety was evaluated based on the occurrence of adverse events, symptoms and physical examination findings, clinical laboratory tests (including complete blood count, blood biochemistry, urinalysis, and coagulation parameters), vital signs (blood pressure, pulse, respiratory rate, and temperature), and 12-lead electrocardiograms (ECGs). The clinical characteristics of adverse events, including their onset time, severity, resolution time, duration, management measures, and outcomes, were recorded. The correlation between adverse events and the study drug was assessed, and the severity of adverse events was graded according to the Common Terminology Criteria for Adverse Events (CTCAE) version 5.0.

### 2.5 Statistical analysis

Pharmacokinetic parameters such as Cmax, ${\text{AUC}}_{0-\infty }$ , AUC_0-t_, t_1/2_, Tmax, λz, and CL/F were calculated using Phoenix WinNonlin (version 8.3), employing the Linear Trapezoidal Linear Interpolation method based on the actual blood sampling times. λz was estimated by log-linear regression of terminal concentrations (ln C vs. time) using the built-in best-fit method with at least three post-Cmax quantifiable points. λz was defined as the negative slope of the regression line. If fewer than three terminal points were available, λz and derived parameters were not reported for that period. The primary pharmacokinetic parameters (Cmax, AUC_0-t_, ${\text{AUC}}_{0-\infty }$ ) were subjected to natural logarithmic transformation, followed by analysis of variance (ANOVA) and 90% confidence interval (CI) analysis. If the 90% CI of the geometric mean ratios of the primary pharmacokinetic parameters (Cmax, AUC_0-t_, ${\text{AUC}}_{0-\infty }$ ) under fed versus fasting conditions completely falls within the 80.00%–125.00% range, it can be concluded that food does not cause a significant difference in the main pharmacokinetic parameters. A mixed-effects model was employed with the natural logarithmic transformation of the pharmacokinetic parameters as the dependent variable. Fixed factors included the dosing period, dosing sequence, and administration method, while subjects were treated as a random effect nested within the dosing sequence. Differences in Tmax were assessed using the Wilcoxon signed-rank test. All statistical analyses were performed using SAS software (version 9.4).

## 3 Results

### 3.1 Demographics of the fasted and fed groups

The baseline characteristics of the participants are presented in Table 1. A total of 80 subjects were screened for the study, with 20 subjects successfully enrolled and randomly assigned. All 20 enrolled subjects completed the entire trial, including drug administration, pharmacokinetic sampling, and safety monitoring. The cohort comprised 10 males and 10 females, with 19 subjects of Han ethnicity and one subject of another ethnic group. The mean age of the participants was 37.8 years.

**TABLE 1:** Baseline demographics.

Baseline parameter	Fasted/Fed (N = 10)	Fed/Fasted (N = 10)	Total (N = 20)
Age, years	38.6 ± 9.06	36.9 ± 6.42	37.8 ± 7.69
Sex, male/female	5/5	5/5	10/10
Weight, kg	61.99 ± 6.79	64.01 ± 6.49	63.00 ± 6.55
BMI, kg/m^2^	22.61 ± 2.10	24.01 ± 2.52	23.31 ± 2.37
Ethnicity, n (%)			
Han ethnicity	10 (100)	9 (90.0)	19 (95.0)
Others	0	1 (10.0)	1 (5.0)

Values are means ± standard deviations, or numbers of subjects (%).

BMI, body mass index.

### 3.2 Pharmacokinetics

**FIGURE 1:**
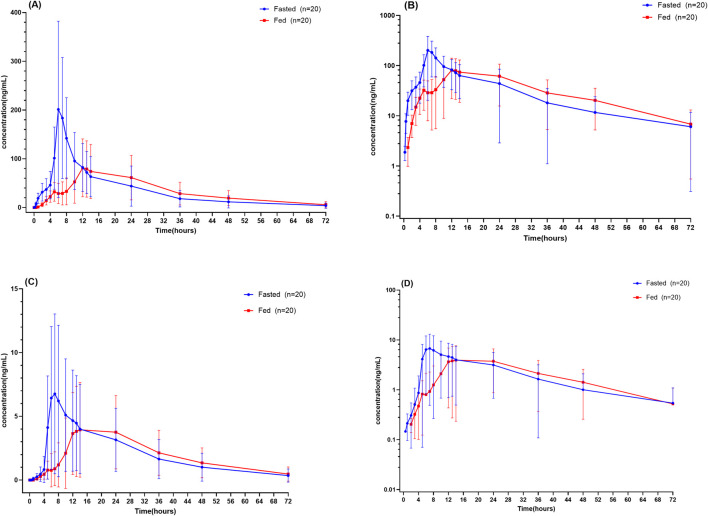
Plasma concentration-time profiles of TSL-1502 and TSL-1502M: **(A)** linear plot and **(B)** semi-log plot of TSL-1502 **(C)** linear plot and **(D)** semi-log plot of TSL-1502M. Data are expressed as mean ± standard deviation (SD).

After a single 200 mg oral dose of TSL-1502 capsules, the primary pharmacokinetic parameters of the parent drug TSL-1502 and its active metabolite TSL-1502M are summarized in Table 2. The mean plasma concentration–time profiles (linear and semi-logarithmic scales) are shown in Figure 1. Comparative analysis of TSL-1502 and its metabolite TSL-1502M between the fed and fasting conditions is presented in Table 3.

**TABLE 2:** Pharmacokinetic parameters of TSL-1502 and TSL-1502M

PK parameter	Fasted (N = 20)	Fed (N = 20)
TSL-1502		
Cmax (ng/mL)	229.99 ± 169.99	96.35 ± 58.30
AUC_0-t_ (h*ng/mL)	2,515.00 ± 1,614.99	2,328.96 ± 1,605.49
${\text{AUC}}_{0-\infty }$ (h*ng/mL)	2,635.63 ± 1738.77	2,479.85 ± 1725.53
t_1/2_(h)	14.07 ± 6.32	14.58 ± 3.76
Tmax(h)	6.50 (5.00, 13.00)	13.00 (5.00, 24.01)
CL/F (L/h)	103.36 ± 50.62	129.81 ± 97.90
TSL-1502M		
Cmax (ng/mL)	8.33 ± 6.11	4.66 ± 3.62
AUC_0-t_ (h*ng/mL)	147.69 ± 129.08	136.94 ± 111.34
${\;\text{AUC}}_{0-\infty }$ (h*ng/mL)	158.77 ± 143.11	163.18 ± 124.76
t_1/2_(h)	14.69 ± 4.81	15.35 ± 3.23
Tmax(h)	7.00 (5.00, 24.00)	14.00 (12.00, 24.01)
CL/F (L/h)	2090.18 ± 1,375.92	2,123.46 ± 1,610.19

Values are mean ± standard deviation, or median (minimum, maximum).

Cmax, maximum plasma concentration; AUC_0-t_, area under the concentration–time curve up to the last quantifiable time; ${\text{AUC}}_{0-\infty }$ , area under the plasma concentration-time curve from time 0 extrapolated to infinity; t_1/2_, terminal elimination half-life; Tmax, time to reach maximum plasma concentration; CL/F, apparent total body clearance.

**TABLE 3:** Ratios for pharmacokinetic parameters of TSL-1502 and TSL-1502M in fed group versus fasted group.

Pharmacokinetic parameters	Ratio (fed/fasted) (%)	90% CI (%)
TSL-1502		
Cmax (ng/mL)	43.42	32.46, 58.07
AUC_0-t_ (h*ng/mL)	86.53	71.23, 105.11
${\;\text{AUC}}_{0-\infty }$ (h*ng/mL)	88.36	73.41, 106.35
TSL-1502M		
Cmax (ng/mL)	52.23	37.63, 72.50
AUC_0-t_ (h*ng/mL)	90.03	72.88, 111.22
${\text{AUC}}_{0-\infty }$ (h*ng/mL)	105.51	93.51, 119.06

CI, confidence interval.

For the parent drug TSL-1502, pharmacokinetic results showed that food delayed the absorption rate of the drug. Under fed conditions, the time to reach the peak concentration (median Tmax: 13.00 h) was significantly longer than under fasting conditions (median Tmax: 6.50 h), while the elimination rate was similar (mean t_1/2_ of 14.07 ± 6.32 h in the fasting group vs. 14.58 ± 3.76 h in the fed group). Under fed conditions, the drug exposure of TSL-1502 was slightly lower compared to fasting conditions, with Cmax significantly reduced. Other pharmacokinetic parameters, including λz and t_1/2_, were similar (Table 2), indicating no significant difference in the elimination rate of TSL-1502 between the two administration conditions. The geometric mean ratios of Cmax, AUC_0-t_, ${\text{AUC}}_{0-\infty }$ for TSL-1502 under fed vs. fasting conditions, along with their 90% confidence intervals, were 43.42% (32.46, 58.07), 86.53% (71.23, 105.11), and 88.36% (73.41, 106.35), respectively (Table 3). Non-parametric statistical comparison of Tmax using the Wilcoxon signed-rank test showed a P-value <0.05, indicating a statistically significant difference between the fed and fasting conditions for Tmax.

For the active metabolite TSL-1502M, pharmacokinetic results showed that under fed conditions, the time to reach the peak concentration (median Tmax: 14.00 h) was significantly longer than under fasting conditions (median Tmax: 7.00 h), while the elimination rate was similar (mean t_1/2_ of 14.69 ± 4.81 h in the fasting group vs. 15.35 ± 3.23 h in the fed group). The drug exposure of TSL-1502M (AUC_0-t_, ${\text{AUC}}_{0-\infty }$ ) under fed conditions was similar to that under fasting conditions, while Cmax was significantly lower in the fed state. Other pharmacokinetic parameters, including λz and t_1/2_, were comparable (Table 2), indicating no significant difference in the elimination rate of TSL-1502M between the two conditions. The geometric mean ratios of Cmax, AUC_0-t_, ${\text{AUC}}_{0-\infty }$ for TSL-1502M under fed vs. fasting conditions, along with their 90% confidence intervals, were 52.23% (37.63, 72.50), 90.03% (72.88, 111.22), and 105.51% (93.51, 119.06), respectively (Table 3). Non-parametric statistical comparison of Tmax using the Wilcoxon signed-rank test showed a P-value <0.05, indicating a statistically significant difference between the fed and fasting conditions for Tmax.

### 3.3 Safety and tolerability

The single oral dose of 200 mg TSL-1502 capsules under both fasting and fed conditions demonstrated good safety and tolerability in healthy Chinese subjects. A total of 20 subjects were included in the safety evaluation. No deaths or serious adverse events (SAEs) occurred during the study, and no adverse events of grade 3 or higher or events leading to withdrawal were reported. All treatment-emergent adverse events (TEAEs) were of grade I or II severity. Detailed TEAEs for the fasting and fed groups are provided in Table 4. A total of eight subjects experienced TEAEs during the study. In the fasting group, four subjects (20.0%; 4/20) reported adverse events, while in the fed group, four subjects (20.0%; 4/20) experienced adverse events. The incidence of adverse events was similar between the fasting and fed groups. All adverse events resolved spontaneously without the need for concomitant medication.

**TABLE 4:** Treatment-emergent adverse events (TEAEs) in the study.

Adverse event	Fasted (N = 20)	Fed (N = 20)
Hypertriglyceridemia	1 (5.0%)	2 (10.0%)
Hyperuricemia	0	1 (5.0%)
Urinary tract infection	2 (10.0%)	1 (5.0%)
Elevated blood bilirubin	0	1 (5.0%)
Paresthesia	1 (5.0%)	0
Nausea	1 (5.0%)	0

The data are presented as n (%).

## 4 Discussion

PARP inhibitors, based on the concept of synthetic lethality, have made milestone contributions to cancer therapy. However, clinical application is limited by hematological toxicities and resistance with long-term treatment. TSL-1502 is the first PARP inhibitor prodrug based on glucuronic acid esterification, which releases the active metabolite TSL-1502M in tumor tissues. Preclinical studies have shown that TSL-1502 exhibits stronger antitumor activity than olaparib and has favorable toxicity profiles (Wang et al., 2021). Currently, TSL-1502 is undergoing Phase I and Phase II clinical trials in patients with solid tumors and breast cancer, respectively. The pharmacokinetics, pharmacodynamics, and safety of TSL-1502 in cancer patients will be further clarified when the results of these trials are published. To date, no studies have evaluated the impact of food on the safety and pharmacokinetics of TSL-1502 capsules.

Previous studies have suggested that oral anticancer drugs are typically administered in the fasting state, as food may increase the bioavailability of the drug by 4–10 times, potentially leading to increased toxicity (Ratain, 2011). Therefore, understanding the effect of food on the pharmacokinetics and safety profile of TSL-1502 can provide guidance for future rational dosing regimens.

This study was conducted in healthy volunteers in accordance with the U.S. Food and Drug Administration (FDA) guidance entitled *Assessing the effects of food on drugs in INDs and NDAs - clinical pharmacology considerations* (US Food and Drug Administration Guidance for industry, 2022), which states that food-effect studies for orally administered drugs are generally performed in healthy subjects when safety permits. Preclinical repeat-dose toxicity studies demonstrated reversible hematologic toxicity and supported an adequate safety margin for the single 200-mg dose administered in healthy volunteers (data on file), thereby supporting the ethical feasibility of conducting the food-effect study in healthy subjects. Because food effects are primarily governed by gastrointestinal physiology, drug solubility, and formulation characteristics rather than tumor biology (Welling, 1996), results from single-dose crossover food-effect studies in healthy volunteers are generally considered predictive for patients unless disease-related alterations in absorption or metabolism or safety concerns necessitate patient-only evaluation. Therefore, evaluating the food effect of TSL-1502 in healthy volunteers provides a regulatorily appropriate basis for informing dosing recommendations in oncology patients.

In this study, 20 healthy subjects were enrolled to evaluate the effect of food on the pharmacokinetics of TSL-1502. Although the sample size was relatively small, the two-period, two-sequence crossover design allowed each subject to serve as their own control, minimizing inter-subject variability and providing sufficient sensitivity to detect differences in key PK parameters (Cmax and AUC). This approach is consistent with FDA guidance on food-effect studies and aligns with the design of similar studies for PARP inhibitors, such as senaparib, fuzuloparib or fluzoparib, which typically enrolled 16–20 subjects to successfully characterize food effects (Meng et al., 2022; Du et al., 2024; Wu et al., 2021). Therefore, the sample size was considered adequate to support the evaluation of the effect of food on the pharmacokinetics of TSL-1502.

This Phase I clinical study evaluated the impact of food on the pharmacokinetics and safety of a single 200 mg oral dose of TSL-1502 capsules in healthy Chinese subjects. The results demonstrated that there were some differences in the pharmacokinetics of TSL-1502 capsules under fasting and fed conditions. The half-life of the parent drug TSL-1502 and the active metabolite TSL-1502M was similar under both conditions. However, under fed conditions, Tmax for TSL-1502 was delayed by approximately 6.5 h, and for TSL-1502M, Tmax was delayed by approximately 7.0 h, indicating that food delayed their absorption.

Under fed conditions, compared with the fasted state, the AUC_0-t_ for the parent drug TSL-1502 decreased by about 13.47%, ${\text{AUC}}_{0-\infty }$ decreased by approximately 11.64%, and Cmax decreased by around 56.58%. For the active metabolite TSL-1502M, AUC_0-t_ decreased by approximately 9.97%, ${\text{AUC}}_{0-\infty }$ increased by about 5.51%, and Cmax decreased by approximately 47.77%. The geometric mean ratios of AUC for both TSL-1502 and TSL-1502M, with their 90% confidence intervals, were within the predefined range of 80.00%–125.00%. Food had a limited effect on AUC of both TSL-1502 and TSL-1502M; however, it substantially reduced Cmax and delayed absorption, while not significantly affecting the elimination half-life. TSL-1502 can be taken with or without food, although the impact of reduced peak concentrations should be considered in subsequent clinical use.

Similar to other marketed PARP inhibitors, such as fluzoparib, olaparib, and niraparib, these drugs can be taken with or without food. Previous studies have shown that a high-fat meal decreases the absorption rate and peak exposure of fluzoparib but does not significantly affect its extent of absorption, and food does not alter the safety profile of fluzoparib (Wu et al., 2021). Similarly, a high-fat meal decreases the absorption rate and peak exposure of olaparib but does not affect the extent of absorption (Plummer et al., 2015). High-fat meals have not significantly affected the absorption extent of niraparib, and it can be taken with or without food (Moore et al., 2018).

In this study, among the 20 healthy Chinese subjects, the incidence of adverse events was 20% (4/20) for both fasting and fed conditions. All adverse events were graded as I or II, and all subjects fully recovered without any sequelae. None of the adverse events required concomitant medication. The safety and tolerability of a single oral dose of TSL-1502 capsules in Chinese healthy subjects was good, with no new safety signals, and food did not affect the safety of TSL-1502. No hematological toxicities were observed in this study, which are commonly associated with other PARP inhibitors (Liu et al., 2023). However, it is important to recognize that this study was conducted in healthy subjects following a single dose of TSL-1502. In cancer patients receiving multiple-dose treatment with TSL-1502, particularly those with underlying hematological vulnerabilities, the incidence and severity of adverse events—including hematological toxicities—may differ, and caution should be exercised when extrapolating these results to the clinical oncology setting.

## 5 Conclusion

Based on the pharmacokinetic results of TSL-1502 capsules in healthy Chinese subjects under both fasting and fed conditions, food has a limited impact on the AUC of the parent drug TSL-1502 and its active metabolite TSL-1502M. The safety profile of TSL-1502 capsules is favorable, and food does not affect the safety of TSL-1502. The analysis suggests that TSL-1502 capsules can be administered with or without food.

## Data Availability

The raw data supporting the conclusions of this article will be made available by the authors, without undue reservation.
